# 2443. Clinical Characteristics and Outcome of Vascular Access Infections in Patients with hemodialysis : A 12 year study

**DOI:** 10.1093/ofid/ofad500.2062

**Published:** 2023-11-27

**Authors:** E U N J U N G LEE, Shinae Yu, Yae Jee Baek, Jongtak Jung, Tae Hyong Kim, Sunin Hong

**Affiliations:** Soonchunhyang University Seoul Hospitals, Seoul, Seoul-t'ukpyolsi, Republic of Korea; Soonchunhyang University Hospital Cheonan, Cheonan, Ch'ungch'ong-namdo, Republic of Korea; Soonchunhyang University College of Medicine, Seoul, Seoul-t'ukpyolsi, Republic of Korea; Soonchunhyang University Seoul Hospitals, Seoul, Seoul-t'ukpyolsi, Republic of Korea; Division of Infectious Diseases, Department of Internal Medicine, Soonchunhyang University Seoul Hospital, Seoul, Seoul-t'ukpyolsi, Republic of Korea; Soonchunhyang university hospital, Cheonan, Ch'ungch'ong-namdo, Republic of Korea

## Abstract

**Background:**

Infection is the second most common cause of death in ESRD (end-stage renal disease) patients. Although vascular access-related infections are directly related to the prognosis and mortality of ESRD patients, related studies are rare.

**Methods:**

The medical records of adult patients admitted due to hemodialysis vascular access-related infections to a 750-bed tertiary medical institution in Seoul, Korea, were retrospectively analyzed from January 1, 2009 to December 31, 2020. Patients younger than 19 years and history of previous vascular access infections in other sites were excluded.

**Results:**

During the 12-years study period, 372 patients were analyzed. According to the vascular access type, there were 290 (78%) of AV graft infections, 34 (9.1%) of AV fistula infections, and 48(12.9%) of tunneled catheter-related infections. Hemodialysis vascular access-related infection occurred at a median (IQR) of 86 (30-132) days in the tunneled catheter, 357 (105-1283) days in AV graft, and 960 (401-2289) days in AV fistula. Bacteremia was present in 152 (40.8%) patients. *Staphylococcus aureus* is the most common organism isolated from blood culture (88, 57.8%). Eighteen patients with methicillin-resistant *S.aureus* (MRSA) bacteremia underwent nasal swabs, and MRSA colonization was confirmed in only 6 of them (6/18, 33.4%). Among the patients without bacteremia, 31 were cultured directly from infected graft tissue or pus.

Metastatic infection was shown in 15 (4%) patients. The most common complication was pneumonia, including septic lung.

Of the 372 patients, 325 (86.7%) underwent surgical treatment. Total excision was performed in 115 (35.3%), partial excision in 178 (54.7%), and incision and drainage in 32 (9.8%). Seventy-nine (24.3%) patients underwent re-operation due to uncontrolled infection.

Three patients died due to vascular access-related infection. Patients who received repeated surgery were at a higher mortality risk (p=0.31).

**Conclusion:**

Tunneled catheter-related infections occurred earliest, while AV fistula infections occurred the latest. *S. aureus* remained the most common causative organism. Patients who underwent revision surgery were associated with death.

**Disclosures:**

**All Authors**: No reported disclosures

